# Enhancing pediatric congenital heart disease detection using customized 1D CNN algorithm and phonocardiogram signals

**DOI:** 10.1016/j.heliyon.2025.e42257

**Published:** 2025-01-25

**Authors:** Ihtisham Ul Haq, Ghassan Husnain, Yazeed Yasin Ghadi, Nisreen Innab, Masoud Alajmi, Hanan Aljuaid

**Affiliations:** a Department of Computer Science, Modeling, Electronics and Systems (DIMES), University of Calabria, 87036, Rende, CS, Italy; b Department of Computer Science, CECOS University of IT and Emerging Sciences, Peshawar, 25000, Pakistan; c Department of Computer Science, Al Ain University, United Arab Emirates; d Department of Computer Science and Information Systems, College of Applied Sciences, AlMaarefa University, Diriyah, 13713, Riyadh, Saudi Arabia; e Department of Computer Engineering, College of Computers and Information Technology, Taif University, P.O. Box 11099, Taif, 21944, Saudi Arabia; f Computer Sciences Department, College of Computer and Information Sciences, Princess Nourah bint Abdulrahman University (PNU), P.O. Box 84428, Riyadh, 11671, Saudi Arabia

**Keywords:** Phonocardiogram, Congenital heart disease, Phonocardiography, Electrocardiogram

## Abstract

Congenital heart disease (CHD), impacting around 1 % of infants worldwide, constitutes a significant healthcare challenge. Early detection is crucial, however constrained by the intricacies of conventional diagnostic techniques such as auscultation and echocardiography. This research presents a tailored one-dimensional convolutional neural network (1D-CNN) for the classification of phonocardiogram (PCG) signals into normal or abnormal categories, providing an automated and efficient solution for congenital heart disease (CHD) diagnosis. The model was trained on a composite dataset consisting of local pediatric PCG signals and publicly accessible dataset. Preprocessing methods, such as low- and high-pass filtering (60–650 Hz), resampling, and noise reduction, were utilized to enhance signal quality. Data augmentation techniques, including chunking, padding, and pitch-shifting, were employed to rectify dataset imbalances and improve model efficacy. Experimental results indicate substantial enhancements, attaining an accuracy of 98.56 %, precision of 98.56 %, F1 score of 98.55 %, sensitivity of 0.98, and specificity of 0.99. The comparative analysis demonstrates the proposed approach's superiority over current methods regarding accuracy and reliability. The research highlights the promise of combining modern signal processing with deep learning for efficient CHD screening. The suggested model exhibits outstanding performance yet, issues like dataset variability and noise persist. Future endeavors involve extending to multiclass categorization and assessing performance across a wider range of medical problems. This study represents a significant advancement in accessible, automated CHD diagnoses, enhancing clinical competence to elevate pediatric treatment.

## 1 Introduction

Congenital heart diseases (CHDs) are a leading cause of morbidity and mortality in both children and adults, presenting either as structural abnormalities existing at birth or as conditions acquired later in life due to factors such as calcium-induced heart valve stenosis, valve leakage, or thrombus formation within the heart. Globally, approximately 1 in every 100 newborns is affected by CHDs, underscoring the urgent need for improved diagnostic strategies [1]. Among these conditions, Tetralogy of Fallot (TOF) is the most prevalent cyanotic CHD, while conditions such as Ventricular Septal Defect (VSD), Patent Ductus Arteriosus (PDA), Atrial Septal Defect (ASD), and pulmonic valve diseases also contribute significantly to the disease burden [2].

Advancements in medical science have considerably increased survival rates among neonates with CHDs, rising from low survival rates in the mid-20th century to approximately 96 % today, owing to pioneering surgical interventions and diagnostic improvements [3]. Early breakthroughs, such as the surgical correction of PDA by Robert Gross and the TOF treatment by Alfred Blalock and Helen Taussig in the mid-20th century, laid the foundation for modern cardiology [4]. These developments were complemented by the introduction of cardiac imaging techniques like echocardiography and arteriography in subsequent decades, facilitating real-time heart monitoring and enhancing diagnostic precision [5]. Despite these advancements, traditional diagnostic methods, including auscultation and echocardiography, often rely heavily on clinician expertise and remain time-consuming and costly. Phonocardiography (PCG), which provides visual representations of heart sounds, has emerged as a valuable diagnostic tool, particularly when paired with electronic stethoscopes to capture and analyze cardiac murmurs. These murmurs, caused by turbulent blood flow or malfunctioning heart valves, are integral to diagnosing CHDs but are often obscured by background noise and variability in data quality [6].

Recent progress in artificial intelligence (AI), machine learning (ML), and deep learning (DL) offers transformative potential for automating CHD diagnosis. Algorithms such as Convolutional Neural Networks (CNNs) and their one-dimensional (1D) variants are particularly effective in analyzing PCG signals, given their capacity to learn and extract features from noisy, high-dimensional datasets. These technologies promise to augment existing diagnostic processes by reducing dependence on clinician expertise while delivering rapid and accurate predictions [7]. This study aims to address the limitations of current CHD diagnostic techniques by proposing a customized 1D-CNN-based approach to classify cardiac sounds as normal or abnormal. The methodology incorporates rigorous preprocessing, including noise filtering and data augmentation, to enhance signal quality and ensure robust model performance. Local and publicly available PCG datasets are utilized, with preprocessing steps such as resampling, windowing, and pitch-shifting applied to address challenges such as data imbalance and noise variability. The proposed model achieves exceptional accuracy, precision, sensitivity, and specificity, highlighting its potential to revolutionize CHD diagnostics. By integrating state-of-the-art AI techniques, this research seeks to bridge the gap between technological innovation and clinical application, ensuring that advancements in automated diagnostic systems can complement human expertise to improve pediatric cardiac care. Future expansions could include multiclass classification of pediatric CHDs and validation of the proposed system across broader medical domains.

## 2 Related work

PCG signals provide secret aspects about the heart's anatomy and behavior, but they are nevertheless widely used in heart sound modeling techniques. Recent years have seen an uptick in the use of the S1, S2, S3, and S4 sounds as diagnostic tools for cardiac disease and injury. An innovative new approach to heart sound analysis combines processing methods with machine learning. An advanced intelligent system for assessing and monitoring heart sounds is urgently required so that they can be transformed into useful clinical informatics tools for the detection of pathological cardiac diseases. This opens the door to the possibility of early diagnosis and treatment, which is crucial for preventing unnecessary deaths. Table 1 shows a comprehensive comparison of state-of-the-art deep learning and machine learning cardiac classification algorithms.

**Table 1:** Existing literature.

Ref	Dataset	AI Model Used	Evaluation Metric	Contribution of Paper	Limitation of Paper
[8]	1000 samples (200 normal, 800 abnormal)	DNN, k-NN	Accuracy: 92.1 % (DNN), 97.4 % (k-NN)	Demonstrated ML- based classification of PCG signals.	Lacks real-world noise handling.
[9]	55 samples (various CHD conditions)	k-NN	Accuracy: 93.2 %	Detected multiple CHD conditions using MFCC features.	Small dataset limits generalizability.
[10]	PhysioNet Challenge 2016 (3099 samples)	1D-CNN, LSTM, Conv1D + LSTM	Sensitivity: 87 %,	Classified PCG signals without feature engineering.	High sensitivity to imbalanced datasets.
[11]	90 PCG and ECG signals	SVM, k-NN, MLP, ML	Accuracy: 92.5 %	Leveraged PSE, WT, and MFCC features for classification.	Limited feature diversity.
[12]	1000 samples, Pascal Dataset B (461 samples), PhysioNet	SVM, k-NN, Random Forest, Naive Bayes, ANN	Accuracy: 99 % (SVM, Random Forest), 76 % (k-NN)	Applied advanced features for heart sound classification.	Computationally intensive feature extraction.
[13]	PhysioNet (3126 recordings)	Random Forest	Accuracy: 92 %	Combined temporal and frequency features.	Not evaluated on noisy data.
[14]	PhysioNet, Pascal Dataset	CNN	Accuracy: 87 % (PhysioNet), 97 % (Pascal Dataset)	Used mel-spectrogram images for classification.	Limited dataset diversity.
[15]	Local hospital dataset (ASD, VSD cases)	SVM	Accuracy: 95.24 %	Focused on ASD and VSD detection.	Limited to specific CHD conditions.
[16]	PhysioNet Heart Sound Database (3153 recordings)	Branched 1D- CNN	Improved Mean Accuracy by 11.84 %	Proposed branched CNN for better classification.	No noise filtering methods used.
[17]	PhysioNet/Challenge 2016 (3126 samples)	k-NN, Decision Trees, Ensemble ANN, LSTM	Accuracy: 91.39 %	Classified PCG using time and frequency features.	Limited interpretability of ensemble methods.
[18]	PhysioNet/Challenge 2016 (3126 samples)	SVM, k-NN, DT, TB	Accuracy: 95.31 %,	Used multidomain features for PCG classification.	Focused only on binary classification.
[19]	Local dataset (86 children: 24 healthy, 62 CHD murmurs)	ANN	Accuracy: 93 %,	Detected murmurs in pediatric CHD cases.	Small dataset impacts generalization.
[20]	Locally captured dataset (1D raw waveforms)	Dense 1D- CNN, Clique 1D-CNN	Accuracy: 96.21 %,	Developed dense and clique CNN models.	Limited external testing.
[21]	103 phonocardiogram samples	VMD, WST	Accuracy: 92.23 %, Sensitivity: 92.4 %,	Applied decomposition techniques for classification.	High reliance on feature engineering.
[22]	UCI repository dataset	CNN	Accuracy: 97 % (binary), 86.67 % (multiclass)	Proposed CNN for binary and multiclass PCG classification.	Dataset imbalances not addressed.
[23]	PhysioNet/Cinc2016	Unique 1D- CNN	Accuracy: 93.21 %,	Classified raw waveforms with 1D- CNN.	Sensitivity below expected for CHD.
[24]	e-general dataset (300 samples)	Fuzzy Inference	Accuracy: 97 %	Used time-domain features and fuzzy logic.	Small sample size limits reliability.
[25]	Freely available dataset	MCS with SVM	Accuracy: 93 %	Combined MCS and SVM for accurate PCG classification.	Lack of noisy data testing.
[26]	69 sounds (37 abnormal, 32 benign murmurs)	ANN	Accuracy: 98 %	Applied ANN for high- accuracy classification.	Very small dataset.
[27]	200 sounds	ResNet	Accuracy: 94.78 %,	Used ResNet and Hilbert envelope features.	Limited feature diversity.
[28]	3200-sound dataset	Segmentation- based models	Accuracy: 79.4 %,	Classified PCG using segmentation techniques.	Low sensitivity for pathological cases.
[29]	PhysioNet	k-NN	97.82 %	Used time-domain features for classification.	Limited comparison with advanced models.

Recent breakthroughs in metaheuristics encompass the Greylag Goose Optimization (GGO) algorithm, which is inspired by the flight patterns of geese, hence improving efficiency in engineering and benchmark tasks [30]. The iHow Optimization Algorithm (iHowOA) emulates human cognitive functions to achieve an enhanced balance between exploration and exploitation in optimization and feature selection [31]. Likewise, the Football Optimization Algorithm (FbOA) employs football tactics to achieve strong performance in intricate, high-dimensional problems [32].

## 3 Materials and methods

**Fig. 1:**
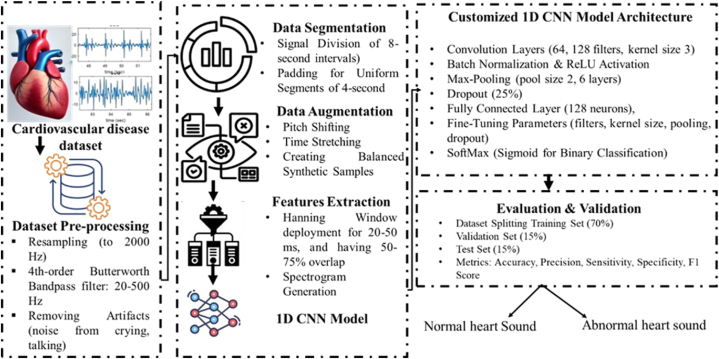
Developed methodology block diagram.

This study employs a structured and sequential methodology for the binary classification of heart sounds. The procedure commences with the procurement of a publicly accessible dataset, which

constitutes the basic data for training and evaluation. This dataset is systematically augmented through successive rounds of methodological improvement to improve robustness. Heart sound waves undergo preprocessing and denoising to enhance quality and remove artifacts that may disrupt analysis. The signals are divided into 8-s intervals to standardize the input and enhance analytical efficiency. A windowing technique is utilized to facilitate the extraction of pertinent signal components, enhancing the input for subsequent processing. Data augmentation techniques, including resampling and synthetic sample generation, are employed to rectify dataset imbalances and enhance training data diversity. These strategies guarantee the generation of uniformly distributed class samples, which is essential for efficient model training. The methodology's essence entails training many 1D Convolutional Neural Networks (CNNs) on the pre-processed and segmented PCG signals as depicted in Fig. 1. These models are refined to enhance network parameters for precise categorization. During the training process, multiple CNN configurations are evaluated to determine the optimal model for identifying heart sounds as either normal or pathological. The methodological procedure, which includes data collection, preprocessing, windowing, data augmentation, and model training, aims to systematically improve classification performance. This thorough methodology guarantees that the final model attains excellent accuracy, rendering it a dependable instrument for automated cardiac sound classification.

A. Dataset

This research employs a composite dataset consisting of publicly accessible and locally gathered cardiac signals. The publicly accessible Physionet dataset [17] comprises high-quality, noise-reduced recordings of heart sounds, ranging from 1 to 1.15 min, encompassing diverse cardiac diseases including ASD, VSD, CAD, Mitral Valve Prolapse, MVR, PVS, SAAS, MS, and AR. Furthermore, locally sourced data from Peshawar Hospitals comprises 143 paediatric PCG recordings of congenital heart disease (CHD) cases collected in 2022–23. The recordings, each lasting 10–12 s, yield 800 samples, comprising 400 normal and 400 aberrant signals in equal distribution. The amalgamation of these datasets guarantees a varied and equitable representation for comprehensive model training and evaluation.

B. Dataset Segmentation and Pre-processing

**Fig. 2:**
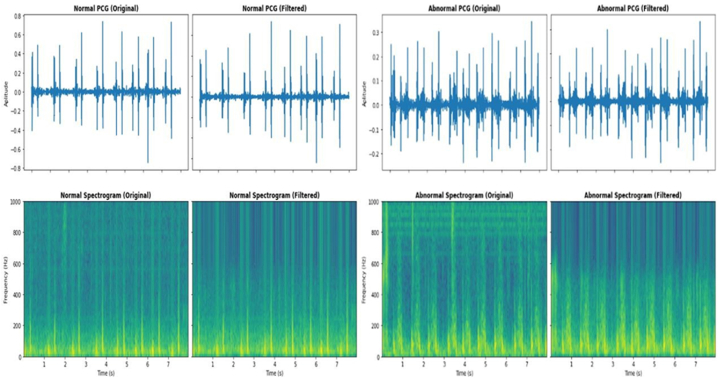
PCG Signal filtration and spectrogram.

To guarantee accurate classification, cardiac sound signal segmentation and preprocessing are crucial to our research. A review of studies found that segmenting signals into 8–12-s periods optimize analysis [10]. This duration was used across the sample for homogeneity. Noise and poor data quality were addressed first in data processing. Signal distortion was caused by crying newborns, ambient conversations, and stethoscope motions [11]. These aberrations could reduce AI model precision, sensitivity, and specificity if unfiltered. To standardize recordings, signals were resampled to 2000 Hz using the Nyquist criterion. This assured all recordings had the same sampling rate. Next, a 4th-order Butterworth band-pass filter with a cutoff frequency of 20–500 Hz removed extraneous frequencies and focused on the important region for paediatric congenital heart disease detection. The frequency range matches medical expert guidelines and detects cardiac signal problems within 3–6 s. High-frequency noise was removed, and key signal components were kept for analysis during preprocessing. The dataset was divided into 4-s chunks to maintain compatibility with previous research methods and improve machine learning model input. Comparing unfiltered and filtered signal spectrograms shows the efficiency of different preprocessing methods as depicted in Fig. 2.

**Fig. 3:**
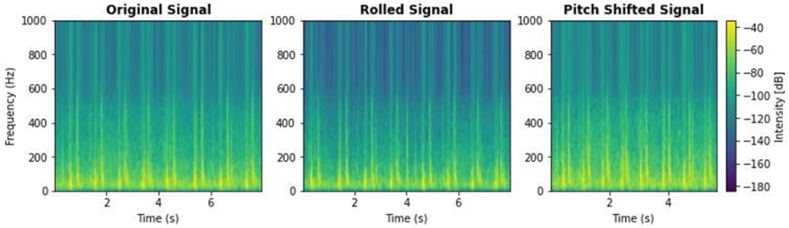
Pcg signal augmentation.

CQ3: Fig. 3 was not cited in the text. Please check that the citation(s) suggested are in the appropriate place, and correct if necessary. Signal Processing and Data Enhancement

Effective signal processing and data augmentation are essential for enhancing the quality and utilization of phonocardiogram (PCG) data. Initially, filtering was utilized to mitigate background (see Fig. 3)

noise, commonly found between 0 and 50 Hz. High-frequency disturbances, such rubbing noises or external sounds exceeding 800 Hz, were attenuated with a bandpass filter. Audacity, a prevalent audio processing application, was employed to apply a filter with a passband frequency range of 60 Hz–650 Hz. This frequency range was selected to maintain the fundamental attributes of cardiac sounds while diminishing extraneous signals. The filter's roll-off was established at 48 dB per octave to ensure effective attenuation of frequencies outside the target range, thus separating pertinent cardiac signals and minimizing interference. Resampling was conducted to standardize the data from various sources. The locally collected dataset, initially recorded at 8 kHz, was resampled to align with the 44.1 kHz sampling rate of publicly accessible datasets. This stage guaranteed uniformity in data representation and rectified possible discrepancies in parameters like sample rates and class distributions, which are essential for dependable model training and evaluation. Data segmentation and augmentation significantly refined the dataset for deep learning applications. The PCG signals were segmented into 4-s intervals to correspond with clinically significant durations. Padding was utilized as needed to maintain consistent segment lengths, capitalizing on the periodic characteristics of cardiac impulses to uphold contextual integrity. Data augmentation methods, including pitch-shifting, were utilized to generate synthetic data variations, so increasing class balance and enhancing model robustness, as illustrated in figure.

3. These augmentation techniques mitigated the constraints of limited datasets and enhanced the AI model's accuracy and sensitivity, particularly for vital applications in healthcare.

D. Features Extraction & Classification

**Fig. 4:**
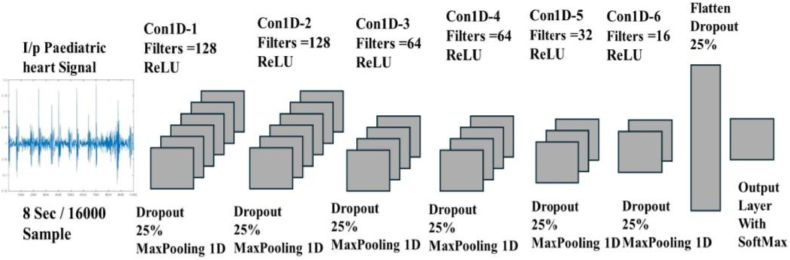
Customized 1D CNN architecture.

Beginning with signal processing and ending with model training and evaluation, this research applies a comprehensive methodology to enhance heart sound classification. When altering the high-frequency component of an audio transmission, pitch shifting is crucial. It increases the frequency range of cardiac sounds from 60-650 Hz to 60–800 Hz, making the S1 and S2 spikes louder and more prominent. As seen in Fig. 4, the modification maintains the initial signal while enhancing the frequency components. The phonocardiogram signals were transformed into spectrograms by use of a Hanning window that had an overlap of 50–75 % and a duration of 20–50 ms. To measure the effect of pitch shifting, a spectrum analyzer was used to examine the frequency spectrum. It shows the results of the investigation, which show an increase in high-frequency spikes, especially in the 250–350 Hz region. Because of its resilience in dealing with noisy datasets, a 1D Convolutional Neural Network (CNN) was used for feature extraction and classification.

Among the features of this design are 64-and 128-filter convolutional layers, batch normalization, and ReLU activation. The kernel size is 3. Dimensionality is reduced by max-pooling layers with a pool size of 2, and overfitting is prevented by a fully connected layer with 128 neurons that incorporates a dropout rate of 0.5. In order to get probabilistic classification results, the last layer employs a SoftMax activation function. The full architecture is depicted in Fig. 4. The 1D-CNN model was trained using a dataset with 15,231 samples, with 70 % of those samples going into training, 15 % into validation, and 15 % into testing. It was fine-tuned using hyperparameter optimization. An F1 score of 98.55 %, specificity of 0.99, sensitivity of 0.98, and accuracy of 98.56 % were all signs of the model's outstanding performance. To facilitate strong multi-class or binary classification, the SoftMax function transformed raw model outputs into probabilities, further refining the classification process. Built on top of Luce's choice axiom, this function makes sure that the results are normalized for probabilistic interpretation, which makes the model more reliable and easier to understand.

**Table:** Pseudocode for Proposed Methodology

1. Let $D=\left\{x_{1},x_{2},x_{3},\ldots .x_{N}\right\}$ represents the Dataset where N = Total no of PCG Signal. Xi = 8 s signal with T = 16000 samples ($f_{s}=2000\;Hz$).
2. Dataset Splitting: Split D into subsets;
Training set: $D_{train}=70\%\;of\;D$
Validation set: $D_{Val}=15\%\;of\;D$
Test set: $D_{test}=15\%\;of\;D$
Dataset Preprocessing
1. Noise Filtering: Apply a 4th-order Butterworth band-pass filter
$x_{i}^{filtered}=ButterworthFilter\left(x_{i},f_{low}=20Hz,f_{high}=500\;Hz\right)$
2. Resampling: All signals to a uniform rate
$x_{i}^{resampled}=Resample\left(x_{i}^{filtered},f_{s}=2000\;Hz\right)$
3. Segmentation: Overlapping segments of 4 s
$x_{i,j}=Segment\;\left(x_{i}^{resampled}.\;T_{segment}=4s\;\right)$
4. Data Augmentation: Perform pitch shifting
$x_{i,j}^{aug}=PitchShift\;\left(x_{i,j},\Delta F\right)$
Features Extraction Via Customized 1D CNN
1. Convolution Layer
$Yl=ReLU\left(Conv1D\left(x_{l-1},Fl,Kl\right)\right)$ where Fl = no of filter, Kl = no of kernel and l = layer index
2. Max Pooling
Yl=MaxPool(yl,P=2) where P = pool size.
3. Dropout Regularization
Yl=Dropout(yl,R=0.25) where R = dropout rate.
4. Flatten & Fully Connected Layers
$Y_{fc}=ReLU\;\left(Dense\left(Y_{flatten},N_{neurons}=128\right)\right)$
5. Output Layer
SoftMax activation for binary classification $\hat{y}=SoftMax\left(Y_{fc}\right)$
Model Training
1. Loss function: Optimize using categorical cross-entropy loss
L=−1N∑i=1N[yi∗log(yˆ)+(1−yi)log(1−yˆi)]
2. Optimization: Update weight θ using Adam Optimizer
$\theta \leftarrow \theta -\eta \nabla_{\theta }L$ where η is learning rate.
Model Evaluation
1. Compute Accuracy, Precision, Recall, and F1-score:
Accuracy=TP+TNTP+TN+FP+FN
Precision=TPTP+FP
Recall=TPTP+FN
F1Score=2∗Precision∗RecallPrecision+Recall
2. ROC and AUC
$AUC=\int_{0}^{1}ROC\left(FPR,TPR\right)d\left(FPR\right)$
Validation and Comparison
1. Validation: Evaluate the model on $D_{Val}\;and$$D_{test}$
2. Comparison: Comparison with baseline Model e.g., SVM, KNN etc etc using accuracy and AUC Metrics.

## 4 Experimentation

This study employs a deep neural network approach to classify cardiac sound data, enabling the differentiation between normal and abnormal heart sounds. The automatic identification of PCG signals constitutes a binary classification problem for computers. A one-dimensional convolutional neural network model is employed to analyze the heart sound data. Utilizing CNNs enables autonomous feature extraction and the processing of high-dimensional data without necessitating compression. The dataset is randomly partitioned into a training set, validation set, and test set. The dataset is partitioned such that the test set comprises 15 %, the validation set constitutes 15 %, and the training set accounts for 70 %. The experiment employs a dataset including 15,231 samples, of which 8076 represent normal PCG signals and 7155 denote abnormal signals. The obtained test data comprises 2285 heart sound samples, whilst the training data is partitioned into training and validation sets including 11,004 and 1942 samples, respectively. The 1D-CNN model undergoes training, validation, and testing initially. The ideal 1D-CNN variant model is subsequently chosen by refining the architecture's filters, kernel size, dropout, and max-pooling layers. The ideal 1DCNN variant model is found by fine-tuning the architecture's filters, utilizing a kernel size of 3 with different units of 128, 64, 32, and 16. The dropout rate is set at 0.25, indicating 25 %, and the max pooling layers have a pool size of 2, with a total of 6 max pooling layers applied. The quantity of maxpooling layers is equivalent to that of hidden layers. The activation function used in this experiment is ReLU; however, due to the binary classification, the ultimate activation function applied is Sigmoid as depicted in Fig. 4.

## 5 Results

This study utilized public and private data. Due to dataset imbalance and inadequacy, initial studies showed that the model's performance was not medically acceptable. Local data had a lot of background noise, and some.wav files had no audio content, making classification harder. Though random variance remained large, pitch-shifting and re-filtering improved the dataset and improved outcomes.

To mitigate these restrictions, the dataset was improved using methods such as fixed-size windowing, sample padding, data augmentation for balance, and meticulous fine-tuning of the network architecture. These enhancements yielded increased stability and precision in classification performance. The model demonstrated optimal performance on the processed PhysioNet dataset, but initially encountered difficulties with the unprocessed raw PhysioNet data. Metrics like accuracy, precision, F1 score, sensitivity, and specificity, as outlined in Fig. 8, Fig. 9 underscore the efficacy of the improved model.

**Fig. 5:**
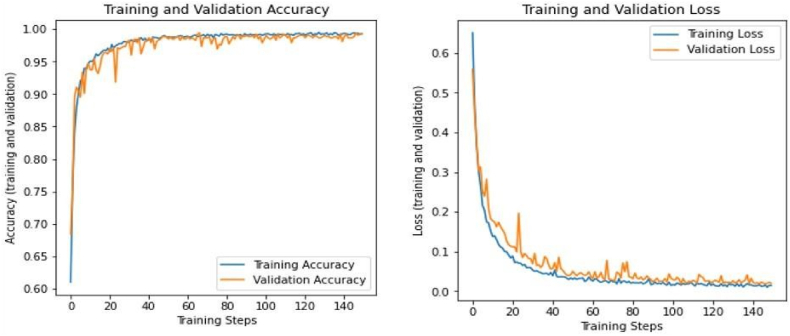
Accuracy and loss of the customized CNN.

**Fig. 6:**
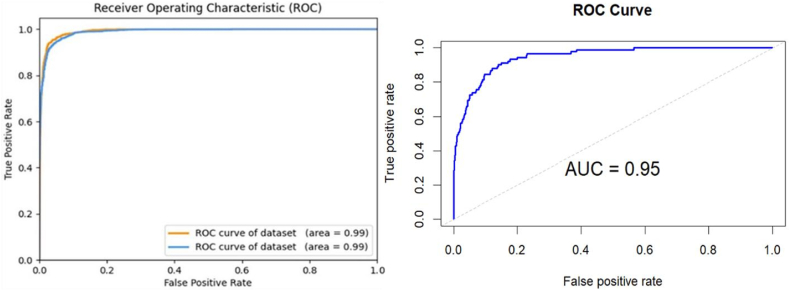
Auc and ROC for the proposed method.

The training and validation phases, depicted in Fig. 5, exhibited consistent enhancements in accuracy and loss with the optimized dataset. Additionally, Fig. 6 illustrate the receiver operating characteristic (ROC) curve and the associated Area Under the Curve (AUC). The AUC of the refined dataset attained an exceptional 0.99, indicating the algorithm's superior proficiency in differentiating between positive and negative samples.

**Fig. 7:**
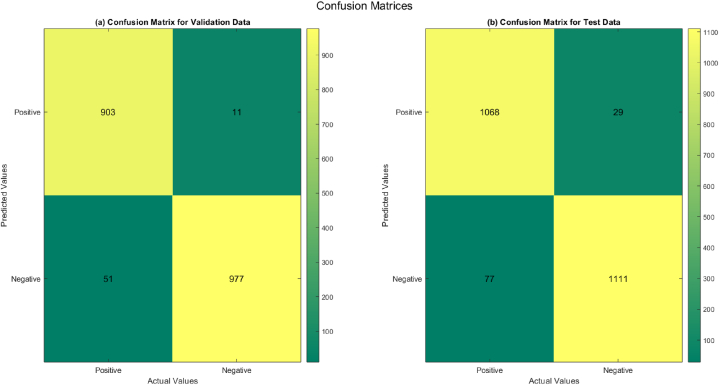
Proposed methodology Confusion Matrix.

These results indicate the model achieved near-perfect accuracy across all dataset categories. Fig. 7 presents the confusion matrix, which elucidates the model's strengths and limitations.

**Fig. 8:**
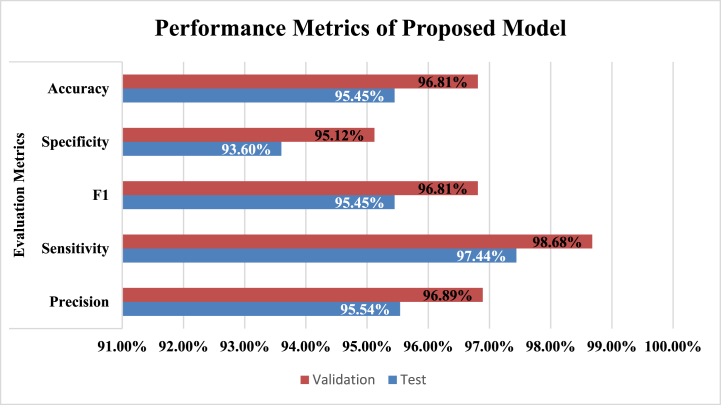
Developed model performance matrix.

**Fig. 9:**
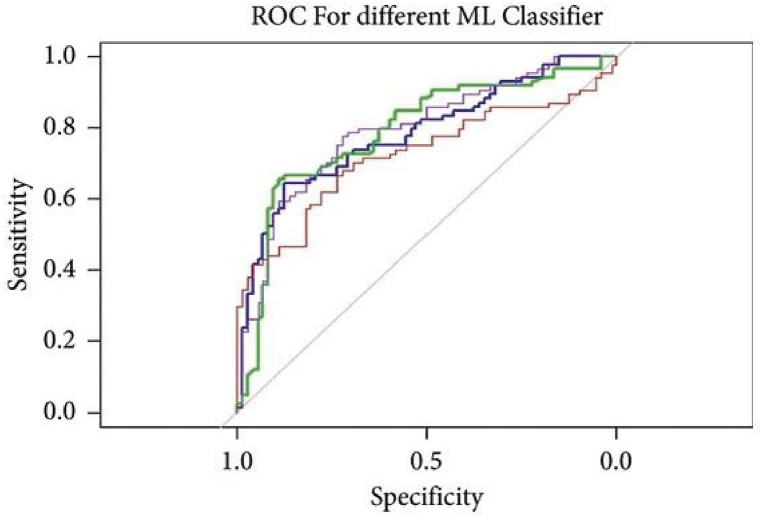
Roc curved for pre-trained model on PCG dataset.

The model accurately recognized 904 normal and 976 abnormal sound samples in the validation dataset but erroneously categorized 12 normal samples as abnormal and 50 bad samples as normal. In the test set, it accurately categorized 1069 normal samples and 1112 abnormal samples, while erroneously categorizing 28 normal samples as abnormal and 76 abnormal samples as normal. These findings highlight the model's robustness and capacity to attain high classification accuracy despite difficulties like dataset imbalance and noise. However, further improvements are needed, particularly in reducing misclassification of abnormal samples, to enhance clinical relevance.

The comparative assessment of the proposed method against the leading methodologies is presented in Table 2. Masun et al. [22] attained a notable 80 % classification accuracy through the analysis of the temporal, frequency, and complexity characteristics of heart sounds. Li et al. [23] similarly segmented the signals and retrieved 497 features from eight distinct categories, including amplitude, time, energy, cyclisation features, frequency, higher-order statistics, cross-entropy, and Cepstrum features. Their implementation of a CNN enabled them to attain an impressive 86 % accuracy with these features. Despite the lower specificity of a prior investigation [33] compared to the suggested study, our technique demonstrates superior performance with minimum effort and complexity. Furthermore, the suggested methodology is independent of heart sound segmentation for R-R intervals and creates only 8-s signal samples, hence enhancing the diagnostic process's speed and efficiency while maintaining significant performance.

**Table 2:** Comparative evaluation of related work based on physionet dataset.

Ref	Model	Accuracy	Sensitivity	Specificity
[22]	LogitBoost + Random Forest + Cost-sensitive classifier	80 %	80 %	81 %
[23]	CNN	86 %	87 %	86 %
[33]	CNN	90 %	90 %	90 %
[34]	Dense-FSNet	86.7 %	94 %	79 %
**Proposed model**	**1D-CNN**	**98.55 %**	**97.55 %**	**93.7 %**
**Pre-trained SVM**	Support Vector Machine	73.08	84.72	61.3
**Pre-trained NB**	Naïve Bayes	64.74	88.89	44.05
**Pre-trained LR**	Logistic Regression	73.08	83.33	64.29
**Pre-trained RF**	Random Forest	79.49	91.04	70.79

The deployment of pre-trained ML models on the final dataset did not yield the anticipated results, unlike the customized one-dimensional CNN model, which clearly demonstrates superior performance in comparison to current models.

## 6 Discussion

The article presents a new method designed to improve the detection of congenital cardiac defects in infants through the analysis of Phonocardiogram (PCG) signals. The methodology depends on a one-dimensional Convolutional Neural Network (CNN). Despite the strong initial performance, challenges arose, including erratic fluctuations, inadequate data, and inconsistencies in sampling. Utilizing advanced techniques such as resampling, chunking, padding, data augmentation, and pitch-shifting, significant enhancements were achieved in algorithmic accuracy, precision, F1 score, specificity, and sensitivity, exceeding 98 %. The detection of murmurs, confirmed through the utilization of the Physionet dataset and local PCG signals, highlights its importance in a clinical setting. The study recognizes some limitations, including the ongoing requirement for medical professional expertise, imbalances within the dataset, and complications arising from noise. The report proposes potential directions for future investigation. These tasks may encompass the exploration of paediatric multiclass classification and the execution of diverse medical diagnostic validations. The algorithm has shown considerable potential in several medical applications, underscoring its versatility and effectiveness in transforming diagnostic methods.

## 7 Conclusion and Future Work

The proposed methodology presents an innovative method for detecting irregularities in the PCG signal, enabling the extraction of clinically relevant cardiac sounds and murmurs, and categorizing the signal into two categories concurrently. This study evaluates the efficacy and adaptability of the proposed strategy utilizing a compilation of local and public datasets comprising both conventional and atypical PCG signals. The Physionet dataset [35] and the continuous PCG signals obtained with our electronic stethoscope constituted the public datasets utilized in this paediatric data analysis. Utilize this integrated dataset to analyze and differentiate the presentation and fundamental outcomes of the proposed method's two information augmentation techniques: piecing and cushioning, and pitch moving. The test findings indicate that the approach attains an exactness of 98.56 %, an accuracy of 98.56 %, an F1-score of 98.55 %, a responsiveness of 0.98, and a specificity of 0.99.

A. Limitation

The technology has the potential to enhance the existing diagnostic process; yet it will still require the experience of medical experts. The study's findings indicate that scientists and engineers will be capable of fully automating the technique as computational capability of computers advances. The authors have recognized the constraints of the proposed methodology in the manuscript. The method still needs the skills of medical professionals, and the study's findings indicate that scientists and engineers will achieve full automation of the technique as computational power advances. Furthermore, we noted that the dataset employed in the study was uneven and insufficient, resulting in erroneous conclusions. The presence of low-frequency noise in the local data may induce random fluctuations in the findings; yet the 1D CNN approach can still yield commendable outcomes despite this interference. The authors have offered insights into the limitations of the suggested strategy; nonetheless, additional research is required to comprehensively comprehend its constraints.

B. Future Work

This research involved the integration of a local dataset with a publicly accessible dataset characterized by differing sampling rates and noise levels. The local data was quite inconsistent. Following meticulous cleaning and filtration, the model trained on that data yields exceptional results. Due to the unavailability of the Paediatric dataset, we limited our analysis to the determination of normal versus abnormal by binary classification. As further local data becomes accessible in the future, this research may be extended to encompass paediatric multiclass classification. In the future, we intend to assess the efficacy of the proposed methodology by applying it to the classification of Oral Squamous Carcinoma cancer OSCC [36,37], diabetes [38,39] breast cancers [40], Brain tumour [41], respiratory disease [42], the prediction of heart diseases [43], to enhance its generalizability.

## CRediT authorship contribution statement

**Ihtisham Ul Haq:** Writing – original draft, Validation, Methodology, Investigation, Formal analysis, Conceptualization. **Ghassan Husnain:** Writing – original draft, Supervision, Investigation, Formal analysis, Data curation. **Yazeed Yasin Ghadi:** Writing – original draft, Visualization, Software, Resources, Methodology, Data curation. **Nisreen Innab:** Writing – review & editing, Validation, Software, Project administration, Investigation, Formal analysis. **Masoud Alajmi:** Writing – review & editing, Resources, Project administration, Investigation, Funding acquisition, Formal analysis. **Hanan Aljuaid:** Writing – review & editing, Visualization, Supervision, Resources, Project administration, Funding acquisition, Conceptualization.

## Data availability statement

The data could be available from the corresponding author upon reasonable request.

## Funding

Princess Nourah bint Abdulrahman University Researchers Supporting Project number (PNURSP2024R54), Princess Nourah bint Abdulrahman University, Riyadh, Saudi Arabia.

## Declaration of competing interest

The authors declare that they have no known competing financial interests or personal relationships that could have appeared to influence the work reported in this paper.
